# Performance of Droplet Digital PCR in Non-Invasive Fetal *RHD* Genotyping - Comparison with a Routine Real-Time PCR Based Approach

**DOI:** 10.1371/journal.pone.0142572

**Published:** 2015-11-12

**Authors:** Iveta Svobodová, Eva Pazourková, Aleš Hořínek, Michaela Novotná, Pavel Calda, Marie Korabečná

**Affiliations:** 1 Institute for Biology and Medical Genetics, First Faculty of Medicine, Charles University in Prague and General Faculty Hospital in Prague, Czech Republic; 2 Department of Nephrology, First Faculty of Medicine, Charles University in Prague and General Faculty Hospital in Prague, Czech Republic; 3 Department of Obstetrics and Gynecology, First Faculty of Medicine and General Faculty Hospital in Prague, Charles University in Prague, Czech Republic; Hospital Authority, CHINA

## Abstract

Detection and characterization of circulating cell-free fetal DNA (cffDNA) from maternal circulation requires an extremely sensitive and precise method due to very low cffDNA concentration. In our study, droplet digital PCR (ddPCR) was implemented for fetal *RHD* genotyping from maternal plasma to compare this new quantification alternative with real-time PCR (qPCR) as a golden standard for quantitative analysis of cffDNA. In the first stage of study, a DNA quantification standard was used. Clinical samples, including 10 non-pregnant and 35 pregnant women, were analyzed as a next step. Both methods’ performance parameters—standard curve linearity, detection limit and measurement precision—were evaluated. ddPCR in comparison with qPCR has demonstrated sufficient sensitivity for analysing of cffDNA and determination of fetal RhD status from maternal circulation, results of both methods strongly correlated. Despite the more demanding workflow, ddPCR was found to be slightly more precise technology, as evaluated using quantitative standard. Regarding the clinical samples, the precision of both methods equalized with decreasing concentrations of tested DNA samples. In case of cffDNA with very low concentrations, variance parameters of both techniques were comparable. Detected levels of fetal cfDNA in maternal plasma were slightly higher than expected and correlated significantly with gestational age as measured by both methods (ddPCR r = 0.459; qPCR r = 0.438).

## Introduction

Non-invasive prenatal diagnosis (NIPD) represents an important area of research since 1997, when cell-free fetal DNA (cffDNA) was detected in maternal plasma and serum for the first time [1]. NIPD tends to partially substitute invasive diagnostic methods indicated nowadays, namely amniocentesis (AMC) and chorionic villus sampling (CVS) [2]. Main advantages of NIPD in comparison with the conventional invasive diagnostic techniques are the absence of negative psychological impact on pregnant women and primarily the elimination of the risk of fetal loss as a consequence of an invasive procedure [3]. The cffDNA, which originates mainly from placental trophoblast [4, 5], is detectable in maternal circulation even before 5^th^ week of gestation [6] and it disappears from circulation quickly after delivery [7]. The content of fetal fraction corresponds to 3–10% of the total cffDNA in plasma [6, 8] depending on gestation age. It is significantly increased in certain cases of pathologic pregnancies (preeclampsia, ectopic placenta, aneuploidy, etc.) [9–11]. Reliable diagnosis based on cell-free fetal DNA is mostly performed after the 10^th^ gestational week.

Currently, the real-time PCR analysis of the cffDNA is broadly applied for fetal RhD status determination from plasma of RhD-negative pregnant women to detect RhD incompatibility between mother and fetus and to prevent the haemolytic disease of the fetus and newborn (HDFN) [12]. HDFN is mostly caused by maternal anti-D antibodies IgG crossing the placenta and destructing the fetal red blood cells [13]. Nowadays, antenatal anti-D immunoglobulin prophylaxis is given to all RhD-negative women. In Europe, for instance, 40% of these women are at no risk of immunization because of carrying RhD-negative fetus. Introduction of the non-invasive fetal *RHD* genotyping using cffDNA prevents the prophylaxis in such cases [14]. The same methodological approach is further routinely implemented for fetal gender detection in families at risk of gonosomal recessive diseases (e.g. haemophilia A and B, Duchenne or Becker muscular dystrophy) and for certain single-gene disorders investigation (β-thalassemia) [15, 16].

Next to the well-established method for cffDNA analysis—real-time PCR (qPCR)—a new quantification strategy, droplet digital PCR (ddPCR), has been recently developed [17, 18].

This new approach is based on portioning of the measured sample into thousands of uniform droplets (separate reactions). Previous dilution of the analyzed sample to the proper concentration so that one template molecule is present per one partition on average (one or zero molecules in most droplets) is necessary. After emulsion PCR, results are obtained by counting the number of positive (one or more molecules of target sequence) and negative (no template) droplets. The correct starting concentration of the template is determined by applying the Poisson statistical analysis to the fraction of positive droplets.

The main advantage of ddPCR in comparison with qPCR is the direct absolute quantification of target nucleic acid molecules without any requirement of calibration curves, moreover ddPCR potentially allows quantification with higher sensitivity. A technical aspect of this new technique makes it an ideal tool for applications like rare event detection or copy number variations estimation, where accurate quantification is required [19, 20]. On the other hand, qPCR still represents most frequently used quantification platform with dynamic range of detection that can exceed 9 orders of magnitude [18].

A few studies have focused on utilization of ddPCR or analogical platforms for non-invasive prenatal diagnosis [21] including fetal chromosomal aneuploidy detection [22, 23] or fetal *RHD* genotyping [24]. However, no test has been introduced in a clinical setting. The most problematic aspect of cffDNA detection from maternal plasma is often low contribution of fetal fraction. There are several possibilities to achieve fetal portion enrichment such as sample size separation [25], maternal background supression [26, 27] or approaches based on particular fetal DNA molecules that exhibit specific methylation patterns distinguishing these fetal sequences from maternal ones [28, 29].

In our study, ddPCR was implemented for fetal *RHD* genotyping from maternal plasma to compare this quantification alternative with qPCR as a golden standard for quantitative analysis of selected cffDNA sequences.

The study was divided into three parts. In the first part, the linearity, detection limit and measurement precision of both methods was evaluated using serially diluted standard human DNA. Four different amplicons (one for *GAPDH* gene and three for *RHD* gene) were examined in all experiments.

In the second part of our study, the plasma samples of non-pregnant RhD-positive women were analyzed to obtain data concerning the performance of both methods on total cell-free DNA in plasma.

The third part of our study focused on examination of plasma samples from pregnant RhD-negative women being at risk to bear the RhD-positive fetus with the goal to compare the performance of both methods on fetal fraction of cell-free circulating DNA in clinical samples.

## Materials and Methods

### Clinical samples

Clinical samples included in our study consist of:

1. 10 peripheral blood samples obtained from RhD-positive non-pregnant volunteers.
2. 35 peripheral blood samples from RhD-negative pregnant women, who were at the risk of RhD incompatibility between mother and fetus.

The written informed consents were obtained from all participants. The study was approved by the Ethical Committee of the First Medical Faculty of Charles University and General Faculty Hospital in Prague (the permission has been issued on 9^th^ June 2014 under No. 62/14).

Fetal *RHD* genotyping using qPCR technology is performed in pursuance of routine diagnostics in cooperation with Department of Obstetrics and Gynecology of the First Faculty of Medicine and General University Hospital in Prague since 2008. Total of 380 samples were processed to date. The RhD status of all women and newborns analyzed in our study was confirmed serologically.

### Sample preparation and DNA isolation

Peripheral blood samples were collected by venipuncture using Vacutainer tubes with EDTA (ethylenediaminetetraacetic acid) to prevent coagulation. The tubes were stored at 4°C and processed within 12 hours. Plasma samples were obtained by centrifugation of the whole blood at 4°C for 10min/2600*g*. The supernatant was transferred to a new tube and centrifuged at 4°C for 10min/14000*g*. Plasma samples were then frozen to -20°C.

For isolation of cell-free DNA from 1ml of plasma, the Qiagen QIAamp Circulating Nucleic Acid kit (Qiagen, Germany) was used according to manufacturer's instructions. In all cases, DNA was finally eluted in 65μl of supplied elution buffer.

### PCR amplification

Concentrations of DNA were in all cases determined as levels of amplification of four regions localised in two genes, specifically one region in reference gene *GAPDH* (glyceraldehyde-3-phosphate dehydrogenase) and three ones in *RHD* gene (D antigen of Rh blood group)–namely in exons 5, 7 and 10. Optimisation by temperature gradient was implemented for each PCR assay and for both methods before initiating the experiments. Each PCR reaction was performed in a 20μl of total reaction volume containing 5μl of isolated plasma DNA. The same primers and probes concentrations were used in both qPCR and ddPCR. The duplex reactions were performed for both methods (*GAPDH*-VIC + *RHD* exon 10-FAM; *RHD* exon 5-FAM + *RHD* exon 7-VIC). All PCR reactions were run in triplicates.

Oligonucleotides for exon 7 of *RHD* and *GAPDH* genes were designed using Primer Express 3.0 software (Life Technologies). The primers and the probe for *GAPDH* amplification were: Forward: 5’-CCCCACACACATGCACTTACC-3’ Reverse: 5’-CCTAGTCCCAGGGCTTTGATT-3’ the probe: 5’-(VIC) TAGGAAGGACAGGCAAC (MGB)-3’. The amplification of *RHD* exon 5 was performed using published sequences of primers and the probe [30]. For amplification of *RHD* exon 7, following primers and the probe were used: Forward: 5’-TGTGCTGCTGGTGCTTGA-3’, Reverse: 5’-AGTGACCCACATGCCATTG-3’, Probe: 5’- (VIC) ACCGTCGGAGCCG (MGB) -3’.

Primers and the probe for amplification of *RHD* exon 10 were commercially supplied by LifeTechnologies. All probes were MGB-labelled except the *RHD* exon 10 probe, which was labelled with TAMRA.

Because of highly polymorphic nature of *RHD* gene, three *RHD* exons are analyzed for elimination of false negative or false positive results in fetal RhD determination. For instance, inclusion of two exons amplified instead of one in diagnostic algorithm reduces the error rate from 1% to 0.01% [31]. *RHD* exons 7 and 10 contain several specific sequences distinguishing *RHD* and *RHCE* genes; in addition exon 5 is included for *RHDψ* variant detection [30, 32]. Thus, combination of these three *RHD* exons is suitable for *RHD* genotype determination and capture of particular *RHD* variants.

#### 1) qPCR

For all qPCR reactions, TaqMan^®^ Gene Expression Master Mix (Applied Biosystems, USA) was used. All qPCR reactions ran on ABI HT7900 real-time PCR instrument (Applied Biosystems) under the following conditions: hold for 10min at 95°C, then 40 cycles of 95°C for 15s and 60°C for 1min. Results were finally processed using SDS Software version 2.4 (Applied Biosystems). The results of qPCR method were recorded as threshold cycle (Ct)–defined as a number of the cycles, in which the measured fluorescence crossed the given threshold (i.e. fluorescence of the background was exceeded). Ct is analogical term to Cq (quantification cycle) recommended to use by MIQE guideline [17].

#### 2) dPCR

In droplet PCR, ddPCR Supermix for Probes (Bio-Rad, USA) was used. The dPCR emulsion was performed by mixing 20μl of prepared reaction mix and 70μl of droplet generation oil (Bio-Rad) using QX100 Droplet Generator (Bio-Rad). For target sequences amplification, Bio-Rad T100 Thermal Cycler (Bio-Rad) was used and following thermal profile was applied: hold for 10min at 95°C, then 40 cycles of 94°C for 30s and 58°C for 1min, 1 cycle of 98°C for 10min and final holding at 12°C. Samples were then loaded into the QX100 Droplet Reader (Bio-Rad). Final evaluation was implemented by QuantaSoft Software version 1.2.10.0 (Bio-Rad) using Poisson statistics. The results were reported as a number of copies per microliter (copies/μl). The average number of droplets accepted in each reaction was 13.791.

### Evaluation of methods

In the first part of the study, both methodological approaches mentioned above were compared using quantitative standard. Standard human genomic DNA of known concentration (TaqMan^®^ Control Genomic DNA, Applied Biosystems) was eightfold diluted and twelve replicates were prepared for each dilution. The performance of both methods was then evaluated using several parameters, such as degree of linearity (R²), detection limit and measurement precision.

In the next step, two types of clinical samples were examined by both methods: The total cfDNA has been examined in plasmas of 10 non-pregnant RhD-positive subjects and cell-free fetal DNA fraction (cffDNA) was studied in plasma of 35 RhD-negative pregnant patients. The partners of all tested pregnant women were positive for *RHD* gene (hemizygous or dominantly homozygous), so there was 50% or 100% probability respectively, of carrying *RHD*-hemizygous fetus endangered by HDFN. Gestational week at the time of sampling was in interval from 12^th^ to 36^th^ (23^th^ week on average).

In case of RhD-positive non-pregnant subjects, all analyzed amplification sites in both genes originate from cfDNA. The content of *GAPDH* sequences corresponds to the total cfDNA concentration in plasma. The same applies to *RHD* sequences in plasma of *RHD*-positive homozygotes, whereas in case of hemizygotes the *RHD* amplification signals reflect only half of the total cfDNA concentration in plasma. Regarding the RhD-negative pregnant women (3), the content of *GAPDH* sequences represents the total cfDNA concentration as well, while the amplification signals of three *RHD* exons originate exclusively from cell-free fetal DNA and correspond to the half of total cffDNA contribution of the hemizygous *RHD*-positive fetus.

Fetal RhD status was considered as positive when the amplification was determined in more than five out of nine *RHD* replicates (three replicates for each examined exon). The sample was considered as RhD-negative when no amplification in any of the nine *RHD* replicates was detected. In case of detection of one to four *RHD* replicates, the sampling and entire analysis was repeated with the same assessment conditions used.

For abnormal maternal haplotype exclusion, the same assays were routinely carried out on DNA obtained from maternal buccal swabs.

### Statistical analysis

Data were evaluated using Statistica 12 software. For normal distribution assessment, the Shapiro-Wilk test was performed. Non-parametric Mann-Whitney test was used for evaluation of differences between coefficients of variation (CV) obtained by both methods during analysis of replicates for standard curves construction. Pearson’s correlation was performed to test correlation between results obtained by qPCR and ddPCR in all assays and also to analyze correlation between fetal fraction of circulating DNA in maternal plasma and gestational age.

## Results

### Linearity, detection limit and measurement precision of ddPCR in comparison with qPCR

1) Performance of both compared methods was assessed using 8-point standard curve ranging from 2 to 0.015ng/μl. The DNA concentrations in the dilution serie were prepared to cover expected levels of cell-free DNA in clinical samples. Each concentration was measured in twelve replicates using amplification signals of four different genomic regions—one in reference gene *GAPDH* and three in *RHD* gene (exons 5, 7 and 10).

At first, the results of both technologies were converted to the same units, namely ng/μl. In case of qPCR, concentrations in ng/μl were determined using standard curve. Regarding the dPCR, the definition of genomic equivalent (GE) was used to convert copies/μl to ng/μl:

1GE/μl = 2 copies/μl = 6,6 pg/μl (diploid genome)

**Fig 1:**
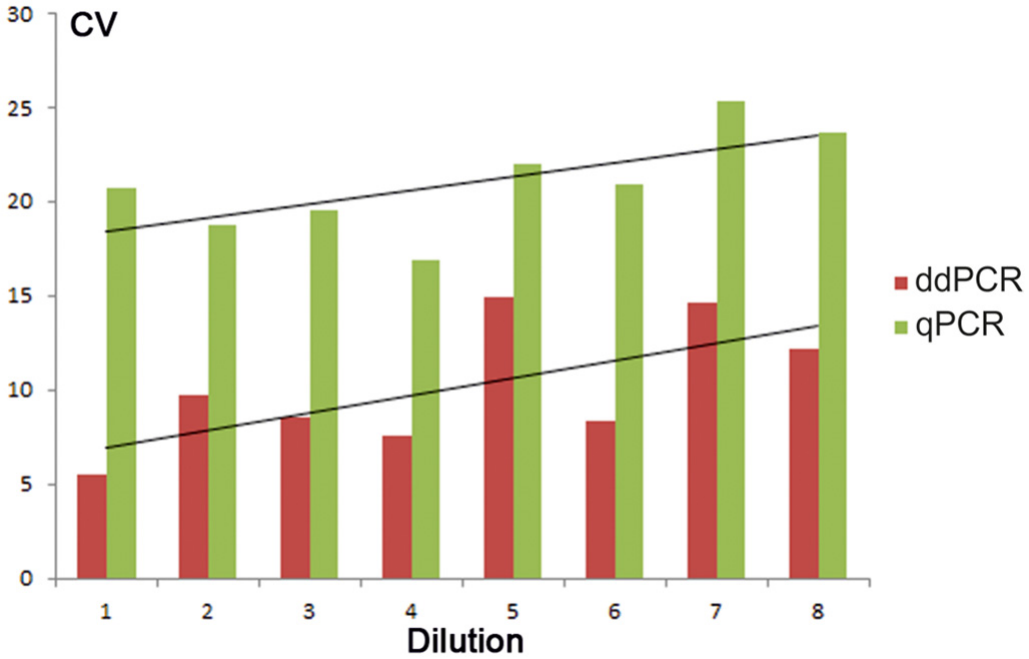
Statistically significant differences between the both techniques coefficients of variation (CV) for standard curve of *RHD* exon 10 replicates. A trend of increasing CVs with decreasing DNA concentrations can be seen in both methods measurement.

Both technologies showed equally low detection limit, all analyzed amplicons were detected in all replicates across all dilution series, including the most diluted samples containing 0.075 ng DNA (11.36 GE) per reaction. The *RHD* genotype of the standard DNA (Taq Man Control Genomic DNA, Applied Biosystems) was determined as homozygous due to the equal PCR amplification parameters obtained for both *GAPDH* and *RHD* exons—equivalent Ct values and numbers of copies/μl respectively were detected in the same standard curve dilutions by all assays. The homozygous *RHD* genotype was respected in all GE calculations.In terms of precision, the ddPCR achieved higher closeness between replicate measurements in general. In case of *RHD* exon 10, the difference was statistically significant (Mann-Whitney, p = 0.0009), as shown in Fig 1.

The both dPCR and qPCR standard deviations (SD) and coefficients of variation (CV) for all standard curves replicates are documented in S1 Table. CV was calculated as follows:

CV = (Standard deviation (SD) / arithmetic mean ) * 100

Excellent linearity has been reached by both strategies above all created standard curves; regression coefficients (R²) were very close to 1: qPCR/dPCR R² - GAPDH (0.9937/0.9927); Rh5 (0.9957/0.9949); Rh7 (0.9951/0.9952); Rh10 (0.9811/0.9953). Standard curves for all assays and methods studied are shown in S1 Fig.

### Comparison of performance of ddPCR and qPCR on clinical samples

**Fig 2:**
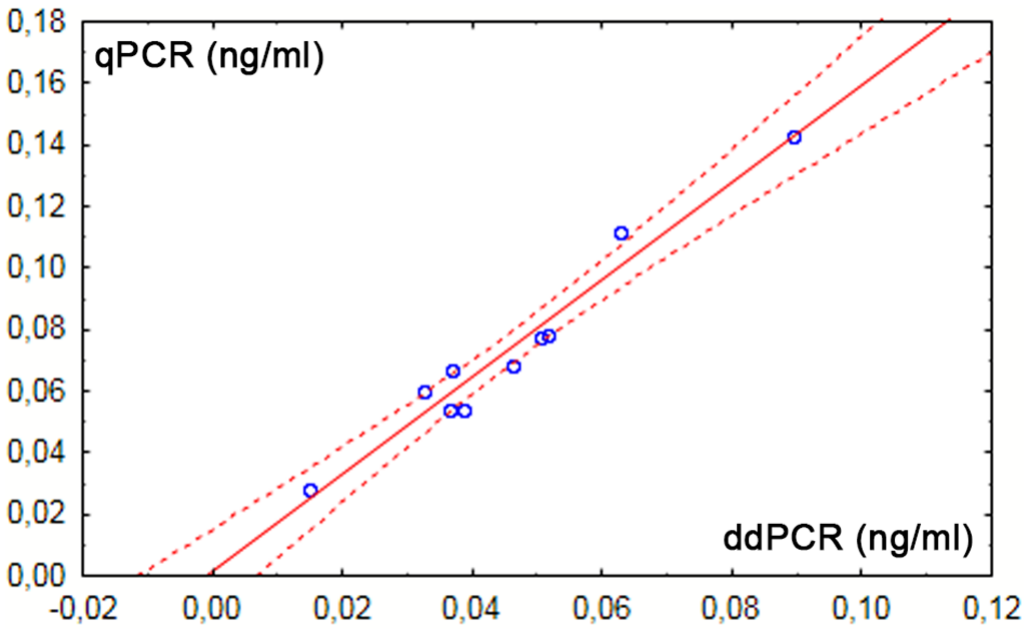
Correlation between measurements of cfDNA concentrations (*RHD* exon 5) by both methods; r = 0.9776, p = 0.000001.

Regarding the plasma cell-free DNA, the correct serologically verified RhD status of all 10 non-pregnant women was determined by both tested techniques. Total cfDNA concentration was measured by *GAPDH* amplification levels. Although absolute DNA concentrations determined using four studied loci slightly varied between two quantification methods, strong correlation between both measurements was found for all assays (Table 1; Fig 2).

**Table 1:** Average concentrations and standard deviations of four analyzed DNA regions measured in plasma of 10 non-pregnant subjects.

Assay	ddPCR		qPCR		Correlation coefficient—r	p-value
	C (ng/ul)	SD	C (ng/ul)	SD		
*GAPDH*	0.0613	0.0182	0.1145	0.0396	0.948483	0.000029
*RHD* 5	0.0460	0.0199	0.0741	0.0323	0.977652	0.000001
*RHD* 7	0.0415	0.0184	0.0389	0.0230	0.970091	0.000003
*RHD* 10	0.0400	0.0187	0.0509	0.0234	0.928794	0.000103

Correlation coefficients show the close agreement between both methods.

Standard deviations among each sample triplicates were slightly lower in case of ddPCR; the differences were not statistically significant (S2 Table).

To compare the performance of both methods on fetal fraction of cell-free DNA circulating in maternal plasma, 35 samples containing cell-free fetal DNA, obtained from RhD negative pregnant patients endangered by RhD incompatibility between mother and fetus, were included to our study and examined by both qPCR and ddPCR. To exclude potential abnormal *RHD* variant of the pregnant women, the DNA samples isolated from buccal swabs of all examined women were analysed using the same assays. In all cases included in our study, no amplification was detected in any of the three *RHD* exons in DNA samples isolated from buccal swabs. Thus, all of the *RHD* amplification signals originated from fetal DNA fraction.

Out of the 35 tested samples, both methods equally found 25 samples to be positive and 10 samples to be negative for fetal RhD status. The results of both approaches were in complete agreement with serologically determined RhD status of the newborns in all cases in which the prenatal non-invasive diagnostics based on cffDNA examination was performed. In all samples containing *RHD*-positive fetal DNA, the amplification was detected in at least seven out of nine *RHD* replicates in both tested methods. The proportion of failed PCR reactions in particular *RHD* exons was similar in both methodologies.

The average absolute concentrations, determined by both methods, were almost the same in the case of cfDNA, but they differ regarding the cffDNA. On the other hand, significant correlations between both techniques have been achieved, as relative quantities of cfDNA and cffDNA were evaluated (Table 2). The variation between replicates was comparable for both ddPCR and qPCR (S3 Table).

**Table 2:** Average concentrations of cfDNA (quantified as *GAPDH* content in all 35 samples analysed) and cffDNA (measured as *RHD* amplification in 25 samples with fetal RhD-positive status) in plasma samples of RhD-negative pregnant women determined by qPCR and ddPCR.

DNA	Average concentration (ng/μl)		Correlation coefficient—r	p-value
	ddPCR	qPCR		
**cfDNA**	0.088	0.089	0.98669	0.000039
**cffDNA**	0.006	0.004	0.93366	0.00069

As correlation coefficients indicate, data measured by both platforms shows close agreement.

**Fig 3:**
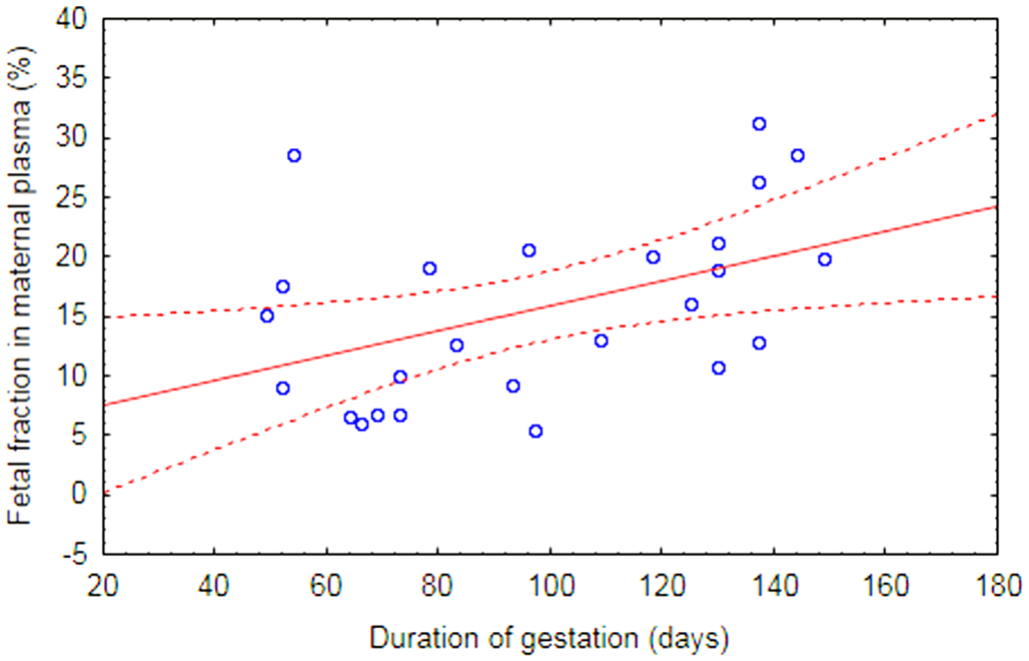
Correlation between gestational age and fetal cfDNA content in maternal circulation measured by ddPCR using *RHD* exon 10*/GAPDH* ratio; r = 0.45936, p = 0.0208.

The proportions of fetal DNA in maternal plasma, determined using *RHD* exon 10*/GAPDH* ratio, were calculated for 25 RhD-positive samples. The average fetal cfDNA fraction represents 15.7% of total cfDNA as measured by ddPCR and 9.8% as measured by qPCR, respectively. Detected levels of fetal cfDNA correlated significantly with gestational age (Table 3, Fig 3).

**Table 3:** Representation of fetal cfDNA fraction in maternal plasma as determined from *RHD* exon 10*/GAPDH* concentration ratios measured by real-time PCR and ddPCR using 25 samples with RhD-positive fetus.

Method	Fetal fraction (%)		Correlation with gestational age—r	p-value
	Average	Range		
**qPCR**	9.8	5.5–31	0.43783	0.0286
**ddPCR**	15.7	1.6–27.7	0.45936	0.0208

## Discussion

This study focused on application of relatively new strategy for quantification of DNA sequences in biological samples—Digital Droplet PCR (ddPCR). The technology was applied to analyze cell-free DNA circulating in plasma including cell-free fetal DNA fraction in pregnant women. The goal of the study was to compare simultaneously ddPCR with qPCR, which is widespread method for DNA quantification routinely used in numerous laboratories and to evaluate the usefulness of ddPCR as methodological alternative for routine application in NIPD.

In the first stage of experiment, the both platforms’ performance was evaluated using serially diluted standard human genomic DNA of the known concentration. Amplification of four genomic regions in two genes–*GAPDH* and *RHD* was assessed. Regarding the detection limit, both methods showed comparable results—all genes were detected in all examined replicates including the most diluted samples (11.36 GE per reaction). Excellent linearity has also been achieved by both ddPCR and qPCR, regression coefficients for all assays’ standard curves neared 1 (S1 Fig). However, droplet PCR displayed lower variability between replicates across all assays and across most of the tested dilutions than qPCR (S1 Table). In case of *RHD* exon 10 assay, the differences were statistically significant (Fig 1).

Consistently with other studies [33, 34], we found ddPCR as the more precise method (lower SDs and CVs respectively, higher regression coefficients of the standard curves). This finding probably reflects the concept of this technology, thus the measuring of the starting concentration of the template. So, the quantification is not influenced by efficiency of the reaction nor by potential inhibitors, unlike the qPCR, where the course of the reaction is evaluated [35].In the next step of our study, the clinical plasma samples were analyzed by both technologies to detect circulating cell-free DNA and its fetal portion in pregnant women. RhD statuses of all tested non-pregnant women, as detected by both methods, corresponded to their serologically determined phenotypes. In terms of pregnant women, 25 out of 35 analyzed samples were equally determined by qPCR and ddPCR as positive for fetal *RHD*, what was in complete agreement with RhD status of newborns detected postnatally by means of serology. Although there was not full match in measurement of absolute concentrations by both systems, the high consensus between compared methods has been reached in terms of relative quantification (Tables 2 and 3; Fig 2).

The implementation of a positive control for the determination of presence of fetal DNA fraction in the sample would be very helpful in this context but unfortunately not easily feasible to date. Differentially methylated regions between fetal and maternal DNA are sometimes used for these purposes, but these loci are often unreliable and methylation is not consistently represented in all cases. The amplification of Y-chromosomal fetal sequences for this purpose will be useful only in one half of pregnancies. Moreover, each additional assay analysed increases the required input volume of the plasma. The amplification of the *GAPDH* sequence serves in the context of our workflow as a control of the quality and quantity of the total cfDNA in examined plasma sample and overall isolation procedure as well. It confirms that the plasma sample is not compromised and that the examination of such a sample will be clinically reliable. The exact quantification of *RHD* sequences in nine replicates provides further control mechanism with regard to potential contamination in *RHD* negative samples and fetal fraction size estimation in *RHD* positive samples.

It is generally known that the precision of the quantification techniques may be lowered with decreasing concentrations of DNA due to stochastic effects. In contrast to the more concentrated standard DNA, variations between cffDNA replicates were the same for both quantification strategies (S3 Table). This effect probably occurred due to Poisson statistics applied by ddPCR for recalculating the number of positive droplets, which slightly limits precision of ddPCR at very low or very high concentrations [20].

The proportional representation of the fetal cell-free DNA fraction from the total cfDNA was slightly higher than expected according to previously published data [6, 8, 29, 36], especially in case of ddPCR. Statistically significant correlations between the week of gestation and fetal cfDNA content in maternal plasma were observed using the both DNA quantification methods (Table 3 and Fig 3). Similar correlation coefficients between gestation age and content of circulating fetal cfDNA in maternal plasma have been found in recent study reporting the use of ddPCR for determination of the size of fetal cfDNA fraction in plasma for NIPD purposes [29]. However, it is clearly visible that gestational week is not the only parameter influencing fetal cfDNA representation in maternal circulation. There are numerous additional factors affecting distribution of cfDNA and cffDNA, such as pregnancy pathologies, especially associated with placenta, that influence trophoblast growth and apoptosis and thereby enhance the fetal fraction [10, 11]. On the other hand, certain maternal health factors, namely physical activity [37] or obesity [38], results in an increased release of cfDNA of maternal origin into the circulation, thus leading to relative portion of fetal DNA decline.

In presented experiments, we document the usefulness of ddPCR for NIPD based on quantification of selected DNA sequences. With regards to the laboratory workflow, ddPCR setup involves more steps in comparison with qPCR—especially enzymatic DNA restriction (if genomic DNA should be analyzed), droplet generation and droplet reading step. Therefore ddPCR is more time consuming than qPCR. Another problematic aspect of ddPCR is represented by correct threshold setting. Using current equipment described in the section Material and Methods, there is no possibility to adjust a uniform automatic threshold for all partitions with identical probe used, so the threshold must be set manually in this case. However, manual threshold setting brings inter-individual and also inter-laboratory variability, especially in analysis of samples with very low DNA concentrations, where the positive droplets layer is formed by only a few partitions with the amplified target sequence. The problems mentioned above may be easily overcome by further development of laboratory equipment. It seems that the method of ddPCR fulfils all prerequisites to be applied after careful validation routinely in clinical NIPD laboratories.

## Conclusion

Digital Droplet PCR in comparison with qPCR has demonstrated sufficient sensitivity for analysis of cell-free fetal DNA and determination of fetal RhD status from maternal circulation. Equivalent results were obtained by both methodological approaches. The both compared methods were able to detect 11.36 GE per PCR reaction. We achieved the results corresponding to the serologically determined RhD status of newborns using cffDNA isolated from 1 ml of maternal plasma with both compared techniques in pregnancies ranging from the 12^th^ to the 36^th^ gestation week. Despite the more demanding workflow, ddPCR was found to be more precise technology, as evaluated using quantitative standard. The precision of both methods equalized with decreasing concentrations of tested DNA samples. In case of cffDNA with very low concentrations, variance parameters of both techniques were comparable. Detected levels of fetal cfDNA in maternal plasma were slightly higher than expected and were probably affected, next to the gestational week, by other clinical factors. Using both compared methods, we found correlations between gestation age and the level of fetal cfDNA in maternal plasma (for ddPCR r = 0.459, for qPCR r = 0.438). These values according to previously published study by I. Manokhina et al. [29] suggest that gestation age is an important but not the only one factor influencing the levels of fetal cfDNA in maternal plasma in physiological pregnancies.

To evaluate fully the benefits of ddPCR technology for NIPD, further studies performed in broader spectrum of laboratories in the context of different application are needed for adoption of this approach into routine practice.

## Supporting Information

S1 Fig Standard curves for experiments on standard DNA for all assays and both methods. (XLSX)

S1 Table Standard deviations and coefficients of variations in experiments on standard DNA. (XLSX)

S2 Table Standard deviations among triplicates, analysis of cfDNA in non-pregnant subjects. (XLSX)

S3 Table Standard deviations among triplicates, analysis of cfDNA in pregnant patients. (XLSX)
